# A Backward Glance at Surgery in 1908

**Published:** 1909-01-02

**Authors:** 


### A Backward Glance at Surgery in 1908.

In calling to mind the items of surgical progress during the past twelve months the most striking feature must be the assurance with which the hæmo-vascular system is now attacked. Trendelenburg has twice operated on the pulmonary artery for embolus; operations on aneurysms by the Matas or allied methods have become fairly common, especially with American surgeons; Wieting, among others, has practised arterio-venous intubation for angiosclerotic gangrene; and, finally, vascular anastomosis has attained a degree of technical perfection permitting successful transplantation of organs such as the kidney from one animal to another. In fact, it may be taken as proved that the reparative processes upon which surgery relies in other tissues are sufficient to integrate wounds in vessel walls even under inherently adverse conditions.

Operations within the thorax, whether upon the great vessels, or upon the lungs or œsophagus, have been greatly facilitated by the employment of positive and negative pressure apparatus, such as those of Roth-Draeger and Sauerbruch, designed to remove or prevent the occurrence of pneumothorax. The apparatus, so far, is expensive and a little cumbersome, but its use will not only extend the field of surgery within the thorax, but also improve the technique of operations for empyema and the like.

The question of cancer, despite an enormous amount of work, has not been very greatly advanced; the International Society of Surgery at Brussels discussed, organ by organ, the present position of operative methods for cancer; many papers have been published indicating an attempt to codify evidence for and against the current theories of causation, and a few further therapeutic measures have been promulgated. Of these, that known as "fulguration," and associated with the name of de Keating-Hart, has attracted some attention. It is applied in connection with open operation under anæsthesia, and consists essentially in the employment of spark discharges of high-frequency currents of very high tension (300,000 volts maximum). The value of the method is still *sub judice*.

Meantime Coley has published a number of successful cases of treatment of inoperable or recurrent sarcoma by the modified "fluid" recently introduced. The new "Coley's fluid" is, to all intents and purposes, a vaccine, for it is a sterilised emulsion of cultures of the streptococcus and B. prodigiosus. Whilst the results thus attained are very encouraging, treatment by *x*-rays and radium seems to be still on its trial; in some forms of malignant disease wonderfully good effects are produced, but there is an element of uncertainty which at times assumes sinister actuality. Handley has introduced a method of dealing with the swollen arm of breast-cancer cases, by forming new lymphatic channels, that promises much relief in this distressing condition.

Steady progress is maintained in the surgery of bones, chiefly in the direction of transplantation of collateral bones, of the prevention of ankylosis by cartilage implantation, and in the actual transference of a portion or the whole of a joint. In abdominal surgery, to mention only one or two points, Moynihan has reported a successful case of complete gastrectomy, and Delagenière ten cases with six successes; Lane indicates an operative mortality for his operation of removal of the colon in the treatment of severe auto-intoxication resulting from obstinate constipation, of 18 per cent.: it cannot be said that his operation has been welcomed by the profession. Mr. Berry's paper to the surgical section of the Royal Society of Medicine on rupture of the intestine was a most important contribution to the subject; he insisted on the adoption of two measures now almost universally employed wherever there is considerable soiling of the peritoneum—namely, the "almost upright" position and massive saline infusion.

Two new methods of diagnosis have attracted considerable notice. The Wassermann or serum diagnosis of syphilis is still upon its trial; but even if it should not prove of the value confidently expected —and in this country the tendency is to require coincident demonstration of the spirochæta pallida—it is still a very interesting practical outcome of all the experimental work on the chemico-molecular content of blood serum.

Turning to treatment, we may mention the extensive adoption of the various forms of hyperæmia connected with the name of Bier. In a great many chronic inflammatory, and even acute suppurative processes passive congestion by ligature or suction has superseded the older ways of attaining similar effects, and a great deal of special apparatus for the purpose is on the market. A boom has been abroad in favour of injections of fibrolysin, and there certainly seems some warrant for its use in conditions such as Dupuytren's contraction and ischæmic palsy; in addition it has been employed to treat all sorts of cicatricial tissue, such as peri-gastric and other peritoneal adhesions, stricture of the urethra, even cirrhosis of the liver, and so on.

The use of atoxyl and anilin-arsenates in the treatment of syphilis has made considerable headway; but it is as yet too early to say whether they will in any way supplant mercury.

Vaccines, with and without opsonic control, are obtaining continually-increasing favour; the caution should be entered, however, that the phenomena of anaphylaxis are likely to stay their indiscriminate use.

In the region of anæsthetics the chief advances have been in the direction of experience with spinal analgesia; but a little has been done in this country, chiefly by the gynæcologists, with scopolamine-morphine. Despite one or two fatalities with the spinal method, it is pretty generally recognised that there is a very definite field for it; the technique is better understood, and the unpleasant after-effects are much less evident in the later series of those who have given it an extended trial. There has been a good deal of discussion during the year as to the responsibility of the anæsthetist in law, and opinion seems to be that he, rather than the operator, is to bear the burden. That the surgeon has quite enough to fear from civil process was brought home by the memorial signed by leading surgeons throughout the world in protest against a verdict in our courts adverse to a surgeon of high repute in an action for *malapraxis* at the end of last year.

A great many points of interest have been raised by the extensive litigation in connection with the Employers' Liability and Workmen's Compensation Acts. To instance one only, it has recently been held that a claimant may properly be required to undergo an operation which promises to remove his disability.

